# Clinical Course of Dysphagia in Patients with Nemaline Myopathy

**DOI:** 10.3390/children9081204

**Published:** 2022-08-11

**Authors:** Yeun Jie Yoo, Bo Kyung Shin, Mi-Jeong Yoon, Seong Hoon Lim, Joon-Sung Kim, Bo Young Hong

**Affiliations:** Department of Rehabilitation Medicine, St. Vincent’s Hospital, College of Medicine, The Catholic University of Korea, 93, Jungbu-daero, Paldal-gu, Suwon 16247, Gyeonggi-do, Korea

**Keywords:** nemaline myopathy, dysphagia, video fluoroscopic swallowing study

## Abstract

Nemaline myopathy (NM) is a rare congenital myopathy, a group of disorders that are clinically and genetically heterogeneous. Infants and children with NM often suffer from recurrent pulmonary infections and swallowing difficulty, leading to malnutrition. However, knowledge about the clinical course and prognosis of dysphagia is limited. In this study, we reported the clinical course of two NM patients suffering from dysphagia. Although tube feeding was required for several months after birth, it was eventually possible to obtain sufficient nutrition with an oral diet. Therefore, dysphagia rehabilitation therapy through a series of evaluations should be considered even in children with severe oral motor dysfunction. Through these cases, physicians should be convinced that the symptoms of dysphagia in children with NM can be improved and be able to encourage their parents by explaining this progress. They have the potential to show improvements in swallowing function and will finally be able to take food slowly but fully orally.

## 1. Introduction

Nemaline myopathy (NM) is a rare congenital myopathy that is a clinically and genetically heterogeneous group of disorders. Pathologically, it is a group of non-dystrophic myopathy conditions in which rod-like structures known as nemaline rods exist in skeletal muscle fibers [1]. Mutations in at least 12 genes are known to cause NM, with the most common mutations found in the genes encoding skeletal muscle alpha actin (ACTA1) and nebulin (NEB) [2,3,4]. Although the clinical severity of NM varies, the typical feature is generalized hypotonia and weakness, especially involving the face, neck flexors, and proximal muscles of the extremities, often including the distal extremities as the disease progresses [2]. In addition, some patients present with severe respiratory muscle weakness, requiring mechanical ventilation at birth, and regular monitoring and management are essential to prevent the risk of death [5].

Infants with NM often suffer from swallowing difficulty, which causes recurrent pulmonary infections and malnutrition. In a previous report of 143 cases of NM, 79 patients of congenital NM had feeding difficulties. In this study, 26% of congenital NM patients complained of food intolerance and required tube feeding such as gastrostomy. In some patients, growth was very limited due to poor intake or chronic illness. Bulbar dysfunction was common in patients with congenital NM, resulting in dysarthria, excessive drooling, and aspiration of oral secretion. Also, anatomical abnormalities in the upper gastrointestinal tract such as pyloric stenosis, achalasia, and gastroesophageal reflux have been reported [5]. Management of these abnormalities and accompanying symptoms is not straightforward. One study reported cases of improvement after Nissen fundoplication for gastroesophageal reflux in those who had persistent vomiting, poor weight gain, and recurrent pneumonia despite conservative treatment [6]. Another study reported a case of progressive dysphagia in a 4-year-old boy with NM who improved after the use of botulinum toxin type A for cricopharyngeal dyskinesia [7]. However, resources on the clinical course and prognosis of dysphagia in congenital NM are limited. In this study, we report the clinical course and characteristics of dysphagia in two children with NM who showed significant improvement with age with dysphagia treatment.

## 2. Case Report

### 2.1. Patient 1

**Figure 1:**
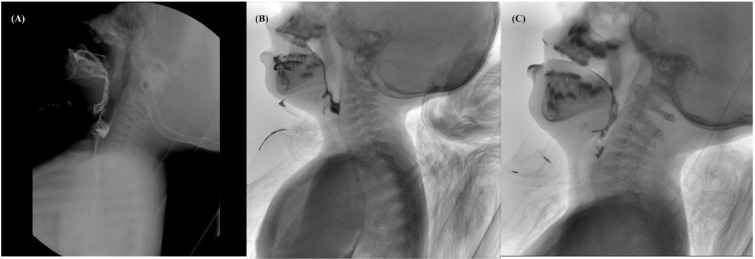
Video Fluoroscopic Swallowing Study (VFSS) of patient 1. (**A**) VFSS at 15 months using pureed foods (IDDSI level 4). More than half of the food remained as oral residue, and significant aspiration of pyriform sinus residue without a cough reflex (PAS 8) was observed. (**B**) VFSS at 32 months using pureed foods (IDDSI level 4). Some collections of oral residues were observed, and food penetration was shown with little contrast in the laryngeal vestibule (PAS 5). (**C**) VFSS at 5 years using pureed foods (IDDSI level 4). The lining amount of the oral residue was observed, the amount of pharyngeal residue was significantly reduced, and no penetration or aspiration was seen (PAS 0).

A 13-month-old boy born at 39 weeks gestation after an uncomplicated pregnancy and cesarean section delivery visited the outpatient clinic for dysphagia and developmental delay. Because of respiratory insufficiency, he was given mechanical ventilation for 3 days after birth. Due to poor sucking reflex, he was fed by nasogastric tube until 7 months of age and then started to eat pureed foods (International Dysphagia Diet Standardization Initiative (IDDSI Level 4)), but it was still difficult to eat. He had clinical features of generalized hypotonia, dysarthria, dysphagia, and facial weakness with an open mouth and drooling. In the Video Fluoroscopic Swallowing Study (VFSS) performed using pureed foods (IDDSI level 4) at 15 months of age, poor lip closure and tongue control without bolus transport were observed, and more than half of the food remained as oral residue. The initiation of pharyngeal swallowing was severely delayed, and minimal laryngeal elevation and epiglottic movement were observed with the aspiration of pyriform sinus residue without a coughing reflex (penetration–aspiration scale, PAS 8) (Figure 1A). We decided to start the dysphagia therapy twice weekly, 30 min per session, while maintaining the diet. Since he avoided oral and facial stimuli and no chewing response was observed when food was given, we massaged the oral structures to facilitate tongue and lip movements and induced a swallowing response by providing passive sensory stimulation. Twice-weekly, 30 min of neuromotor electrical stimulation for each session was used to strengthen the anterior neck muscles. We applied a biphasic symmetric pulsed current with a phase duration of 300 µs an interphase duration of 100 µs, and a pulse duration of 700 µs, resulting in a frequency of 80 Hz. The intensity of the current started from 4.5 mA and was adjusted to 6~7 mA until laryngeal elevation was palpated or observed by the therapist. A pair of electrodes was placed horizontally on the skin just above the hyoid or thyroid notch [8,9]. In addition, parents were educated on oral and facial massage that can be performed at home. At 34 months of age, a muscle biopsy revealed nemaline rod myopathy, and NEB gene mutation was confirmed by genetic examination.

At the follow-up, VFSS was performed using pureed foods (IDDSI level 4) at 2 years (32 months), anterior to posterior bolus transport, and some collections of oral residues were observed. Although the initiation of pharyngeal swallowing was still delayed, the laryngeal elevation and epiglottis movement was improved, and food penetration was shown with little contrast in the laryngeal vestibule (PAS 5). (Figure 1B). At the age of 3 years (44 months), the VFSS was conducted with cheese (IDDSI level 6). Although bolus preparation was prolonged, and some collection of oral residues remained, the initiation of pharyngeal swallowing was significantly improved. Further improvements in laryngeal elevation, epiglottis movement, and laryngeal vestibular closure were observed, with mild penetration in pureed foods (IDDSI level 4, PAS 3) and thin liquid (IDDSI level 0, PAS 2). While chewing, the stability of the neck and jaw was insufficient, and the oral motor muscles were weak. Therefore, we performed Shaker exercise and buccinator and masseter muscle strengthening exercises using resistance during the therapy sessions. In VFSS at 4 and 5 years of age, slices of strawberry (IDDSI level 6) were tried, and bolus preparation and transport efficiency continued to improve. A trace amount of oral residue was observed. The amount of pharyngeal residue was markedly reduced, and no penetration or aspiration was seen in any of the diets evaluated, including pureed foods (IDDSI level 4, PAS 0) and thin liquid (IDDSI level 0, PAS 0) (Figure 1C). He is now 6 years old and can eat uncooked side dishes (IDDSI level 6). As the dysphagia improved, the caregiver reported reduced mealtime and that the patient had become able to eat safely.

### 2.2. Patient 2

**Figure 2:**
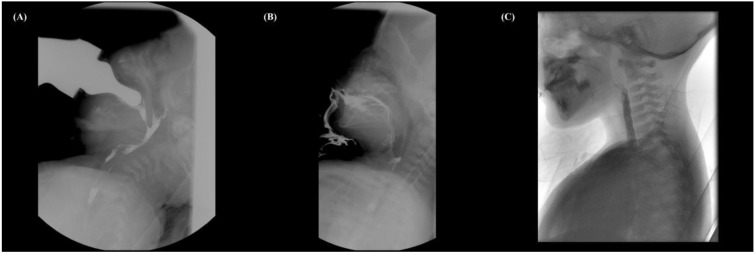
VFSS of patient 2. (**A**) VFSS on the 42nd day after birth with a bottle of milk (IDDSI level 0). The initiation of pharyngeal swallowing was severely delayed, the soft palate elevation was reduced, and repeated aspiration into the airway without a cough reflex (PAS 8) was observed. (**B**) VFSS at 10 months using pureed foods (IDDSI level 4). Food spilled beyond the chin due to poor lip closure, and minimal bolus preparation or transport occurred. Laryngeal elevation and epiglottic movement were partially improved so that there was no aspiration or penetration (PAS 0), with some pharyngeal residue. (**C**) VFSS at 2 years (35 months) using a scrambled egg (IDDSI level 5). Although she exhibited weak, slow, and prolonged mastication, she has demonstrated safe and efficient swallowing with a well-formed bolus.

A girl born through normal delivery without complication at 39 weeks gestation was admitted to the outpatient clinic on the 37th day after birth due to decreased sucking power and cyanosis during bottle feeding. When she breathed, stridor was heard, oxygen saturation dropped to 85% intermittently, and mild laryngomalacia was suspected upon laryngoscopy examination. Her clinical features included generalized hypotonia and micrognathia. At 42 days of age, a VFSS test performed using a bottle of milk (IDDSI level 0) showed a severe delay in initiation of pharyngeal swallowing and decreased soft palate elevation. In addition, there was the minimal laryngeal elevation and epiglottic movement. Most of the contrast remained in the pyriform sinus, with a narrow column of contrast remaining in the laryngeal vestibule, resulting in repeated aspiration into the airway without a cough reflex (PAS 8) (Figure 2A). Because she suffered from repeated pneumonia and sepsis, she relied primarily on nasogastric tube feeding. We started dysphagia rehabilitation therapy twice a week for 30 min or longer. She was observed to hyperextend her neck when eating and exhibited a weak sucking reflex that was not coordinated between sucking, swallowing, and breathing. We trained the parents to maintain her semi-upright posture while supporting her head and neck. A sucking reflex was induced by gently stroking the palate using the therapist’s finger, and a small amount of mild bolus was used to provide a coordinated sucking experience [10].

A follow-up VFSS test was performed using milk (IDDSI level 0) and oatmeal (IDDSI level 4) at 5 months of age. She frequently refused to suck on the nipple of the bottle and spat out most of the milk. She showed a similar pattern when oatmeal was given with a spoon. There was no significant improvement in VFSS findings. Because she exhibits food intolerance and minimal chewing responses when given food, we massaged the oral structures to promote tongue and lip movements and provide a variety of sensory stimuli to elicit a swallowing response. Additionally, neuromotor electrical stimulation was used to strengthen the anterior neck muscles in the same method as in patient 1. When swallowing was induced by using pureed foods, pharyngeal swallowing occurred at a faster rate than before with a reduced cough reflex. At 10 months, when the VFSS was performed using pureed foods (IDDSI level 4), food spilled due to poor lip closure and minimal bolus preparation or transport occurred, resulting in minimal clearance of the bolus in the oral cavity. Laryngeal elevation and epiglottic movement were partially improved so that there was no aspiration or penetration with pureed food; however, there was some pharyngeal residue (Figure 2B).

A muscle biopsy performed at another hospital at 16 months of age confirmed nemaline rod myopathy, but the genetic test results were unknown. In the VFSS test conducted at 20 months with porridge (IDDSI level 5), improvement of lip closure was noted, and a trace amount of contrast outlined the interlabial space. However, minimal bolus preparation or transport was still observed, which results in minimal clearance of the bolus in the oral cavity. A small amount of safe pharyngeal swallowing was observed. Her food intolerance began to improve. She no longer needed a nasogastric tube to thrive. During the dysphagia therapy, we trained her to compensate for poor bolus transport with a head lift, continuous tongue movement, and strengthening exercises using a spoon.

At the age of 2 (35 months), in the follow-up VFSS using a scrambled egg (IDDSI level 5), mastication was first observed, though it was weak, slow, and prolonged. Significantly improved bolus preparation and transport were observed, with some collection of oral residues. The initiation of pharyngeal swallowing was delayed, but only mild penetration (PAS 2) was observed when drinking juice (IDDSI level 0) (Figure 2C). She is now 5 years old and still takes about an hour for each meal, but is able to obtain nutrition fully orally.

## 3. Discussion

Our patients showed characteristics consistent with typical congenital NM. To date, there is no curative therapy for NM. However, most patients in this clinical classification are known to slowly achieve motor milestones with continued physical therapy [5]. It is noteworthy that, in our case, the swallowing function in these patients can also be significantly improved. Although maintenance of the gastric tube was required for several months after birth, obtaining sufficient nutrition with an oral diet was eventually possible.

In a previous review on muscle disorders affecting dysphagia, patients with NM reported moderate oral and pharyngeal phase impairment but no esophageal phase impairment, consistent with our findings. The muscles of the pharynx and larynx may be affected, and the masseter and pterygoid muscles are weak [11]. The characteristics of dysphagia in our patients are (1) poor lip closure; (2) decreased bolus preparation and transport due to impaired tongue and jaw movement; (3) delayed initiation of pharyngeal swallowing, reduced laryngeal elevation and epiglottis movement, and incomplete laryngeal vestibule closure resulting in penetration and aspiration into the airways; and (4) pharyngeal residue due to decreased pharyngeal contraction. Most of the problems improved significantly, but the last remaining problems were decreased tongue and labial strength and dysfunctional jaw movement. Parent education was required to consume a sufficient amount of food, and continuous oromotor strengthening and mastication training were conducted through rehabilitation therapy.

Children with NM who have dysphagia often experience weight loss and frequent pneumonia and remain on gastric tube feeding. Several previous case reports have reported that children with dysphagia require invasive treatments such as botulinum toxin injection or surgical treatment [6,7]. However, since detailed clinical information on the improvement of dysphagia through non-invasive treatment in children with NM has not been reported, it is difficult to predict the prognosis or plan treatment. As seen in our cases, once the amount of aspiration starts to decrease in the serial VFSS evaluation, active and persistent dysphagia rehabilitation should be considered even in children with severe oromotor dysfunction. They have the potential to show improvements in swallowing function, and they will finally be able to take food slowly but thoroughly orally. Physicians, therapists, and parental support for ongoing treatment and patient maintenance of mealtimes can lead to valuable results.
